# Using Real-Life Data to Strengthen the Education of Pharmacy Technician Students: From Student to Research Assistant

**DOI:** 10.3390/pharmacy8020062

**Published:** 2020-04-08

**Authors:** Bjarke Abrahamsen, Rikke Nørgaard Hansen, Marianne Bjørn-Christensen, Tina Druskeit, Charlotte Rossing

**Affiliations:** 1 Department of Research and Development, Danish College of Pharmacy Practice, Milnersvej 42, Hillerød 3400, Denmark; RNH@pharmakon.dk (R.N.H.); cr@pharmakon.dk (C.R.); 2 Department of Education, Danish College of Pharmacy Practice, Milnersvej 42, Hillerød 3400, Denmark; mb@pharmakon.dk (M.B.-C.); tdo@pharmakon.dk (T.D.)

**Keywords:** social pharmacy, pharmacy technician student, education, pharmaceutical care, patient safety, pharmacy practice research, medication beliefs

## Abstract

This commentary is based on the experience of teaching and observations of how pharmacy technician students can expand their perspective on patient safety by using real-life student-gathered patient data collected from community pharmacies. Pharmacy technicians in Denmark work extensively with counselling on the safe and efficient use of medications. Final-year pharmacy technician students can take the elective course in Clinical Pharmacy in Community Pharmacy, which targets the students who wish to work in depth with patient communication and quality assurance in counselling. One assignment that forms part of the course is for students to collect data about patients’ beliefs about medications. Teachers’ observations suggest that when students gather and work with their own data, they change their perspective on patients’ beliefs about medications. It also strengthens the students’ awareness of their responsibility for ensuring patient safety and contributes valid data to research in pharmacy practice.

## 1. Pharmacy Technicians Contributing to Patient Safety and Efficient Use of Medications through Education and Practice

Community pharmacies in Denmark contribute to the healthcare system by offering a variety of pharmacy services to support the patients’ optimal use of medications [1]. In Denmark, the number of community pharmacies is lower than the European average, with 8.6 community pharmacies per 100,000 citizens compared to the European average of 32 community pharmacies per 100,000 citizens [2]. In Denmark, community pharmacies must be owned by a pharmacist holding a five-year MSc university degree in Pharmacy. Throughout the opening hours of a community pharmacy, a pharmacist must be available in person at the main pharmacy and in close contact for pharmacy branches related to the main pharmacy. The largest group of personnel at Danish community pharmacies is pharmacy technicians who hold a three-year degree, as outlined below. The allocation of responsibilities between pharmacists and pharmacy technicians is decided at each pharmacy according to the Danish Healthcare Quality Program [3] with the owner having the overall responsibility. However, some pharmacy services, such as the new medicine service and medication review, must be undertaken by a pharmacist. Pharmacy technicians have direct patient contact; they counsel patients at community pharmacies and deliver some pharmacy services, e.g., the inhaler technique assessment service. Representative mapping of pharmacy technicians counselling activities in Danish community pharmacies shows that 58.9% of all patients served by a pharmacy technician received counselling [4]. Counselling was provided to all groups of patients; patients getting prescription drugs, over-the-counter drugs, presenting with a symptom, or requesting a non-medical product. Furthermore, pharmacy technicians identified drug-related problems (DRP) for 17.8% of all pharmacy customers and counselled them accordingly, solving or partly solving 70.4% of all identified DRP. The study investigated 17,682 customers served by 76 pharmacy technicians from 38 community pharmacies over a duration of five days [4]. According to the survey on the scope of practice for pharmacy technicians from 2017, pharmacy technicians from other European countries have similar work activities, such as receiving prescriptions, investigating the dose and type of drug, and counselling patients about their medicines. The survey shows that British and Portuguese pharmacy technicians are most comparable with Danish pharmacy technicians [5].

Another study based on answers to a questionnaire from 313 Danish community pharmacy technicians showed that they rank the task of providing customers with information as one of their top three task preferences [6]. Around 68% of pharmacy technician students, as well as qualified pharmacy technicians, hold a position within a community pharmacy [7]. Because most pharmacy technicians working in community pharmacies in Denmark have close communication with patients, they require strong communication competences. This commentary reports experiences with strengthening patient communication skills through an assignment for final-year pharmacy technician students complementing existing courses on safe and efficient use of medications. It reports how the students were prepared for the assignment, how they worked with real patient data, and how the teachers observed changes in the pharmacy technician students’ perspective of patients’ use of medications.

## 2. Preparing Pharmacy Technicians in Terms of Patient Safety

The Danish Pharmacy Technician program is a three-year education program equivalent to 180 ECTS (European Credit Transfer System) points. The academic part, which takes place at the Danish College of Pharmacy Practice, corresponds to 85 ECTS points. Students spend a total of 23 weeks taking eight courses at college. Each course lasts two or three weeks, and there are three courses in year one, two in year two, and three in the final year of the program. The practical part of the education program corresponds to 95 ECTS points and takes place at a community pharmacy, where students are employed full-time and are part of the pharmacy schedule except when taking courses at college.

The overall objective of the Danish Pharmacy Technician program is to educate and provide students with the tools and knowledge that enable them to assess and provide professional information and improve patient safety while working in a systematic, methodical, and quality-conscious way to meet needs of the society. The competences that Danish pharmacy technicians gain are professional knowledge, an ethical approach, and a sense of accountability, where consideration of medication user conditions is essential for their practice.

Pharmacy technician students in their final year can choose the elective course in Clinical Pharmacy in Community Pharmacy equivalent to 13 ECTS points. The course focuses on patient safety, patient counselling, rational pharmacotherapy, and awareness of the role of community pharmacies in the healthcare sector. The course particularly targets the pharmacy technician students who wish to work extensively with patient communication and quality assurance of the counselling related to medication safety and rational pharmacotherapy.

The course comprises a total of 16 learning objectives, with four learning objectives associated with the pharmacy technician students’ responsibility for providing patient safety and supporting safe and efficient use of medications. The learning objectives relevant to the focus of this commentary are to:

- Become aware of the responsibility of community pharmacies to ensure patient safety;
- Be able to discuss patient safety in the context of community pharmacy practice;
- Acquire the relevant theory and methods for practicing pharmaceutical care and patient safety;
- Be able to counsel using the patient’s perspective on health and disease.

The teaching approach is to establish a link between students’ own practical experience and the acquired theory. This is achieved through students working alone, in groups, and at plenary sessions discussing the subjects based on their experience.

## 3. Educational Intervention—Using Real-Life Data

Part of the course on clinical pharmacy in community pharmacy is a specific assignment using student-gathered data. This is to target the teaching and augment students’ perspectives on issues such as awareness of how different behaviors can affect adherence to medications and how pharmacy staff can support the patient’s adherence. The assignment has the following learning objectives:

- To become aware of how patients’ concerns and requirements influence initiation and continuation of their use of medications (student level);
- To collect data on concerns and necessity for patients’ use of medications (course level).

The assignment is centered around students’ collection of data based on patient interviews uncovering patients’ beliefs about their medications. In the assignment discussed in this commentary, the students were introduced to the theory of the Beliefs about Medicines Questionnaire [8]. The students discussed how a patients’ view on necessity and concerns about their medicines could affect their initiation, continuation, and overall adherence to their treatment. The students were prepared for the task through an introduction to the questionnaire before their community pharmacy placement. The students were requested to collect and register data from patients, one questionnaire per patient, during their seventh community pharmacy placement. All data were initially recorded by the students on paper before being electronically registered, also by the students, using a web-based survey tool. In 2018, SelectSurvey was used, and in 2019, Microsoft Forms were used. During their community pharmacy placement, the students received e-mail reminders about data collection and could contact a teacher if they had any questions.

### 3.1. Outline for the 2018 Approach

- Registered students for the course: 99
- Number of e-mail reminders: 1
- Number of students to contact a teacher: 0
- Students were asked to collect data from 10 patients
- Returned questionnaires: 231 (equaling 2.3 datasets per student)

### 3.2. Outline for the 2019 Approach

- Registered students for the course: 70
- Number of e-mail reminders: 3
- Number of students to contact a teacher: 0
- Students were asked to collect data from 6 patients
- Returned questionnaires: 311 (equaling 4.4 datasets per student)

## 4. Qualified Teaching Using Patient Data

Following the data gathering, the results were discussed once students were back at college. Starting with a plenary session, a researcher presented the results and an analysis of the data. The students were encouraged to ask questions about the results and to share their immediate reflections. This session was followed by group discussions where students worked with the results and discussed how to use their new knowledge to generate questions which could be used in a counselling situation to identify patients’ perspectives on their use of medications. In the final plenary session, the groups presented their work and received feedback from the teachers and other students.

During the students’ subsequent eighth community pharmacy placements, they presented the results of the study to their pharmacy mentor together with suggestions of how to use the results in counselling situations to identify the patients’ perspectives on their use of medications. The future use of the results and suggestions was left to the community pharmacy to decide as part of their ongoing quality improvement.

Listed in Table 1 is the summary of what the teachers observed during the teaching in relation to both learning objectives for the course and for the assignment.

## 5. Discussion

Data collection by students was introduced in 2015 to strengthen the students’ perspective on the patients’ use of medications and to complement the teaching of the subject already established. By having a questionnaire, students get the experience of asking questions that they may not have the time or courage to ask during a busy working day at the pharmacy. Since 2018, patients have been asked to give their consent for the use of data for research purposes. Both the questionnaire and the introduction were improved to facilitate and support data collection. All students collected and registered data, but not all students registered the intended number of completed questionnaires. From 2018 to 2019, the questionnaire was further improved, and the requested number of questionnaires (one per patient) was reduced from 10 to 6. The changes in the questionnaire, introduction and the possibility for support may be some of the reasons for the increase in the number of completed questionnaires per student from 2.3 to 4.4.

Obtaining student-gathered data from patients representing several community pharmacies and discussing the data in groups as well as during plenary sessions has, according to the teachers’ observations, facilitated a change in the students’ perspective of patients’ beliefs about medications. The teachers’ observations highlighted the fact that students’ perspectives went from being limited to demonstrating a more empathic understanding of patients’ different views, which can have a direct effect on the patients’ use of medication. By generating questions and listening to the patients’ answers, students demonstrated how to use the knowledge they gained to meet the learning objectives for both student and course levels. During the students’ seventh community pharmacy placement, e-mails were sent to remind students to collect and register data. From 2018 to 2019, the total number of returned questionnaires increased from 2.3 to 4.4, possibly due to the increase in reminders from one to three. Students could also contact a teacher with questions about the assignment, but no students used this option. The course is the students’ first experience with collecting data for both teaching and research in community pharmacy practice. This adds an increased focus on preparing students to collect valid data. Discussing data collection and introducing students to the theory before the community pharmacy placement is one way to prepare students.

Over the past 20 years, involving MSc in Pharmacy students in pharmacy practice research has proven fruitful for both students and researchers [9]. During a six-month community pharmacy internship, MSc in Pharmacy students contributed to several published studies through data collection using questionnaires and interviews with patients, pharmacy staff, and general practitioners [9].

In 2016, the International Pharmaceutical Federation (FIP) developed a set of Workforce Development Goals (WDG) at the Global Conference on Pharmacy and Pharmaceutical Education, where milestones were established for impactful global development of pharmacy and pharmaceutical science education [10]. Thirteen goals were set under three clusters: academy (focus on schools, universities, and education providers); professional development (focus on the pharmaceutical workforce); and systems (focus on policy development, governmental strategy and planning, and monitoring systems) [10].

This commentary demonstrates that the Danish Pharmacy Technician program supports the three WDG set for the academia. Under the academic capacity, the Danish education of pharmacy technicians is regulated at the governmental level, including pharmaceutical sciences and clinical practice to support the skills required of qualified pharmacy technicians. The education supports a quality-assured needs-based education and training system. Finally, the training in community pharmacy practice and early career development are supported by offering postgraduate training on the core skills needed to work as a pharmacy technician in a Danish community pharmacy [10].

## 6. Limitations

The students were introduced to collection and registration of data, but no actual control of possible selection bias, errors in data registration, or how the students interacted with patients took place. The evaluation of the teaching and the effect of the students gathering patient data are based on the teachers’ observations, leading to a possible bias where the teachers favor a more positive outcome. A formal evaluation of the students’ outcome by researchers would have been of great value and is a possibility in the courses in the coming years.

## 7. Conclusion

Involving pharmacy technician students in data collection at the patient level, according to the teachers’ observations, has strengthened the students’ awareness of their responsibility to ensure patient safety. The students collected the data that can be used for teaching as well as research and, when discussing the data, the teachers observed an advanced level of understanding from students of how optimal counselling can uncover and accommodate patients’ concerns and beliefs about the necessity of using medications. Furthermore, the teachers’ observations showed that by generating questions and interventions, the pharmacy technician students demonstrated how to counsel and acknowledge patients’ different beliefs about medications. Overall, this commentary reports the experiences from an assignment where students, in addition to their formal academic training, have to relate to real-life data about the patients’ perspective of using medications. The teachers’ reflections indicate that students working with the data they have collected themselves could be a method to strengthen the students’ perspective on optimal patient counselling.

## Figures and Tables

**Table 1:** Summary of the teachers’ observations regarding learning objectives.

**Learning Objectives for the Course**	**Summary of Teachers’ Observations**
Become aware of the responsibility of a community pharmacy to ensure patient safety.	The students became aware that different patients have different needs to discuss and learn how to use medications.
Be able to discuss patient safety in the context of community pharmacy practice.	The students worked extensively with data in groups as well as in plenary sessions. Furthermore, the students presented and discussed the results as part of their subsequent community pharmacy placement.
To acquire the relevant theory and methods to practice pharmaceutical care and patient safety.	Through the work with data, subjects like pharmaceutical care and patient safety were put into the context of the students’ work at a community pharmacy.
Be able to counsel using the patient’s perspective on health and disease.	By generating questions and interventions, the students demonstrated how to counsel and acknowledge patients’ different beliefs about medications.
**Learning objectives of the assignment**	
Become aware of how patients’ concerns and requirements influence initiation and continuation of their use of medications (student level).	The students became aware of patients’ concerns and requirements for their medications by comparing their data with pooled data. The students learned first-hand how patients sometimes think differently from pharmacy technicians. Most patients had different views on medication than the students. For example, the students experienced that patients often expressed more concern and less necessity about using medications compared to what the students would expect when they looked at the collected data. The students learned that by using appropriate counselling they can uncover patients’ concerns and requirements for medications. This can be done by showing an interest in the patient, listening to the patient, and using questions to target the patients’ beliefs about medications, thus facilitating patient safety. Examples of the questions generated include: “Some patients don’t consider this medication necessary; how do you feel about having to take the medication?” ”What thoughts do you have about this medication?” “Do you know why your doctor has found it necessary for you to use this medication?” “Some patients are concerned about the side effects. Do you have concerns?” “Are you managing to take a tablet every morning?” “How important is it for you to take your medication every day?” “Do you know the consequences of not taking your medicine every day?” Finally, the students also saw the benefits of a follow-up to support the patient. Through the follow-up, a community pharmacy can uncover patients’ concerns and support their use of medications.
Collect patient data on concerns and necessity during community pharmacy placement (course level).	The students registered data during their community pharmacy placement according to the instructions in the questionnaire. These data proved to be of high quality and valid for use in research projects.
